# RGX Ensemble Model for Advanced Prediction of Mortality Outcomes in Stroke Patients

**DOI:** 10.34133/bmef.0077

**Published:** 2024-11-26

**Authors:** Jing Fang, Baoying Song, Lingli Li, Linfeng Tong, Miaowen Jiang, Jianzhuo Yan

**Affiliations:** ^1^ Faculty of Information Science and Technology, Beijing University of Technology, Beijing 100020, China.; ^2^ Department of Neurology, Xuanwu Hospital, Capital Medical University, Beijing, China.; ^3^ The Beijing Institute for Brain Disorders, Capital Medical University, Beijing 100069, China.

## Abstract

**Objective:** This paper aims to address the clinical challenge of predicting the outcomes of stroke patients and proposes a comprehensive model called RGX to help clinicians adopt more personalized treatment plans. **Impact Statement:** The comprehensive model is first proposed and applied to clinical datasets with missing data. The introduction of the Shapley additive explanations (SHAP) model to explain the impact of patient indicators on prognosis improves the accuracy of stroke patient mortality prediction. **Introduction:** At present, the prediction of stroke treatment outcomes faces many challenges, including the lack of models to quantify which clinical variables are closely related to patient survival. **Methods:** We developed a series of machine learning models to systematically predict the mortality of stroke patients. Additionally, by introducing the SHAP model, we revealed the contribution of risk factors to the prediction results. The performance of the models was evaluated using multiple metrics, including the area under the curve, accuracy, and specificity, to comprehensively measure the effectiveness and stability of the models. **Results:** The RGX model achieved an accuracy of 92.18% on the complete dataset, an improvement of 11.38% compared to that of the most advanced state-of-the-art model. Most importantly, the RGX model maintained excellent predictive ability even when faced with a dataset containing a large number of missing values, achieving an accuracy of 84.62%. **Conclusion:** In summary, the RGX ensemble model not only provides clinicians with a highly accurate predictive tool but also promotes the understanding of stroke patient survival prediction, laying a solid foundation for the development of precision medicine.

## Introduction

According to statistics, one in 6 people worldwide will suffer a stroke, with over 13.7 million individuals experiencing a stroke annually and 5.8 million dying from it. Comparatively, cancer sees approximately 9.7 million cases; cerebral stroke cases rank second only to cancer. It is estimated that over 80 million people globally have survived a stroke [1]. Therefore, early identification of stroke types and corresponding interventions and treatments substantially impact patient prognosis and quality of life. Specifically, cerebral stroke accounts for the largest proportion of stroke cases. This study focuses on the prognosis of ischemic stroke, which constitutes about 60% of all strokes, thus making the findings highly applicable.

Acute ischemic stroke refers to the sudden loss of blood flow to a specific brain area, resulting in the loss of neurological function. It is caused by the formation of a thrombus or embolism that obstructs the cerebral blood vessels supplying a specific brain region [2]. During vascular occlusion, damage to the core brain area is irreversible. However, in the penumbra region, the brain loses function due to reduced blood flow but does not sustain irreversible damage [3,4]. Given the vast number of individuals affected by cerebrovascular diseases, the application of artificial intelligence (AI) in this field is extensive. In studies diagnosing ischemic stroke patients, 3 machine learning models were developed based on different evaluation metrics to classify thrombus images of patients with occult cancer. These models have become automated decision support tools for predicting occult cancer as a cause of ischemic stroke [5,6]. The team led by JoonNyung Heo developed and validated a machine learning model for predicting patients with occult coronary artery disease and evaluating the long-term outcomes of acute ischemic stroke patients. They utilized an extreme gradient boosting (XGBoost) model to predict any degree of coronary artery disease, achieving an area under the curve (AUC) of 0.763 upon validation [7]. The team led by Thaddeus J. Haight employed deep learning algorithms for the automatic prediction of new images, gradually implementing this system in clinical practice [8]. Thomas Raphale Meinel’s team developed and validated a multivariable prognostic model to identify futile recanalization therapies in patients undergoing intravenous thrombolysis and mechanical thrombectomy. They evaluated 32 variables using *K*-means clustering and employed a gradient boosting decision tree model for prediction, achieving an AUC of 0.87. This model demonstrated moderate overall performance and can aid in shared decision-making [9]. One-fifth of patients with ischemic stroke experience embolic strokes of an undetermined source. Machine learning algorithms can refine the distinction between cardiac and noncardiac embolism. This approach was applied to a separate group of patients to determine predictive mechanisms using L1 regularization, XGBoost, random forests, and multivariate adaptive splines, with random searching used to determine the model’s parameters. This model can accurately distinguish between cardioembolic and noncardioembolic strokes [10]. AI can be utilized in cerebrovascular disease medical imaging for assisted detection, prediction, and treatment support. During detection, AI can help segment vascular lesions with relatively regular shapes, such as spherical or ellipsoidal aneurysms, aiding doctors in diagnosis and treatment [11]. In clinical vascular image analysis, machine learning methods, compared to manual expert analysis or invasive quantitative techniques, achieve over 70% sensitivity and specificity for image segmentation, disease risk prediction, and case quantification [12]. For specific patients, such as those with end-stage renal disease, machine learning can predict mortality and cardiovascular disease in dialysis patients [13]. In routine clinical practice, AI software platforms can expedite the detection of infarctions and cerebral hemorrhages. Algorithms and neural networks have been analyzed for these cerebrovascular diseases as well [14].

Among the complications of cerebrovascular diseases, acute kidney injury is common. Machine learning methods can be used to develop models that predict the risk of acute kidney injury in critically ill patients with acute cerebrovascular disease [15]. The functional outcomes of acute ischemic stroke are a major concern for patients, their families, and their doctors. Machine learning can be utilized to predict these functional outcomes [16]. Similarly, machine learning models can be employed to predict subsequent vascular events 6 months after a mild ischemic stroke in Chinese patients [17]. For the prognosis of ischemic stroke, machine learning models can explore clinical factors associated with long-term swallowing recovery in patients with poststroke dysphagia [18].

## Results

In this study, we propose a new integrated model, the RGX model, which aims to improve the accuracy and robustness of the classification task. Specifically, the RGX model is composed of several base classification models, including random forest, gradient boosted decision tree (XGBoost), logistic regression (LR), and decision tree models. In this study, preliminary experiments were conducted to compare the performance of each base model, and then the experimental results selected the top 3 best-performing models, the random forest model, the XGBoost model, and the decision tree model, integrating the RGX model in this study. Finally, the dataset was divided into training and test sets according to the standard machine learning division method, and the experiments showed that the integrated RGX model performs better in all evaluation indexes.

### MIMIC-IV dataset training

This study utilized data from Medical Information Mart for Intensive Care IV (MIMIC-IV), from which 3,647 patients were initially identified. After extracting the data of patients with an ischemic stroke diagnosis from the database that met the International Classification of Diseases (ICD-9 and ICD-10) criteria, 1,274 patients were ultimately included in the model validation stage after the exclusion of certain criteria. The data are summarized in the Table.

**Table.** Comparison of demographic and clinical characteristics and outcomes between survival and death groups

Variables	Survival group (*n* = 926)	Death group (*n* = 347)	*P* value
Age, years	67.0 [56.8–77.0]	74.0 [63.0–82.0]	<0.001
Body weight, kg	82.0 [69.2–97.3]	76.3 [64.8–91.6]	<0.001
BMI, kg/m^2^	29.0 [25.7–33.7]	28.4 [24.7–33.6]	0.216
Male, *n* (%)	521 (56.3)	180 (51.9%)	0.161
Systolic blood pressure, mmHg	140.0 [120.0–159.0]	140.0 [99.0–163.0]	0.498
Diastolic blood pressure, mmHg	74.5 [64.0–85.0]	70.0 [60.0–84.0]	0.008
Oxygen saturation, %	95.3 [85.6–97.6]	91.3 [81.0–97.0]	<0.001
GCS	12.0 [3.0–15.0]	13.0 [6.0–15.0]	0.306
Smoking	99 (10.7)	30 (8.6)	0.281
Hyperlipidemia, *n* (%)	505 (54.5)	184 (53.0)	0.630
Diabetes, *n* (%)	310 (33.5)	140 (40.3)	0.022
Atrial fibrillation, *n* (%)	326 (35.2)	155 (44.7)	0.002
Coronary heart disease, *n* (%)	369 (39.8)	145 (41.8)	0.530
Heart failure, *n* (%)	190 (20.5)	128 (36.9)	<0.001
Pneumonia, *n* (%)	124 (13.4)	88 (25.4)	<0.001
Respiratory failure, *n* (%)	172 (18.6)	122 (35.2)	<0.001
Renal disease, *n* (%)	17 (1.8)	15 (4.3)	0.012
Sepsis, *n* (%)	66 (7.1)	64 (18.4)	<0.001
Laboratory tests			
White blood cells, K/μl	9.8 [8.0–12.1]	10.8 [8.3–13.8]	<0.001
Red blood cells, K/μl	3.6 [3.1–4.2]	3.3 [2.9–3.8]	<0.001
Platelets, K/μl	212.0 [170.1–269.3]	212.2 [150.1–271.2]	0.092
Neutrophils	7.8 [5.4–11.0]	8.4 [6.2–12.7]	0.003
Hematocrit, %	32.5 [28.6–37.6]	29.9 [27.0–34.5]	<0.001
APTT, s	31.9 [28.0–48.3]	33.7 [28.3–50.5]	0.070
PT, s	13.0 [11.9–14.7]	13.8 [12.6–15.8]	<0.001
INR	1.2 [1.1–1.3]	1.3 [1.1–1.5]	<0.001
D-dimer, ng/ml	1,310.0 [1,233.0–4,055.0]	1,445.0 [1,233.0–4,055.0]	<0.001
Blood urea nitrogen, mg/dl	17.9 [13.5–24.5]	26.6 [17.1–39.5]	<0.001
Bicarbonates, mmol/l	24.0 [22.0–26.0]	25.0 [22.0–28.0]	0.011
Sodium, mmol/l	139.2 [137.0–14.4]	139.3 [136.9–142.2]	0.452
Calcium, mmol/l	8.7 [8.3–9.0]	8.5 [8.2–8.9]	<0.001
Potassium, mmol/l	4.1 [3.9–4.3]	4.1 [3.9–4.4]	0.034
Triglycerides, mg/dl	119.1 [84.0–168.0]	114.0 [83.0–172.5]	0.489
Mechanical thrombectomy, *n* (%)	13 (1.4)	8 (2.3)	0.261
Alteplase, *n* (%)	115 (12.5)	58 (16.7)	0.046

APTT, activated partial thromboplastin time; BMI, body mass index; GCS, Glasgow Coma Scale; INR, international normalized ratio; PT, prothrombin time

By plotting the features with correlation coefficients greater than 0.15 in pairwise plots, as illustrated in Fig. S1, it was determined that, with the exception of the red blood cell and hematocrit features, which demonstrated a discernible linear relationship, the remaining features did not exhibit a significant correlation with one another. In light of the intricate and particular nature of the clinical data, we employed SPSS technology to undertake a comprehensive examination of the correlation between the clinical data, with the objective of ascertaining the existence of a statistically significant association between the features. The *P* value serves to evaluate the reliability of statistical outcomes, functioning as a crucial indicator of data quality. In general, when the *P* value is less than 0.05, the results are considered statistically significant, indicating that the observed differences or associations are unlikely to have occurred by chance. The Table presents the distinctive characteristics of the survival and mortality groups. Continuous variables are expressed as medians and interquartile ranges, while categorical variables are expressed as numbers and percentages. By means of statistical analysis, it was possible to ascertain which variables exhibited significant differences between the survival and death groups. This provided a crucial foundation for clinical decisions and interventions. In conclusion, the comparative analysis of the characteristics of the different groups provides valuable insights into potential key factors affecting survival, further underscoring the significance of SPSS technology in clinical data analysis.

**Fig. 1.**
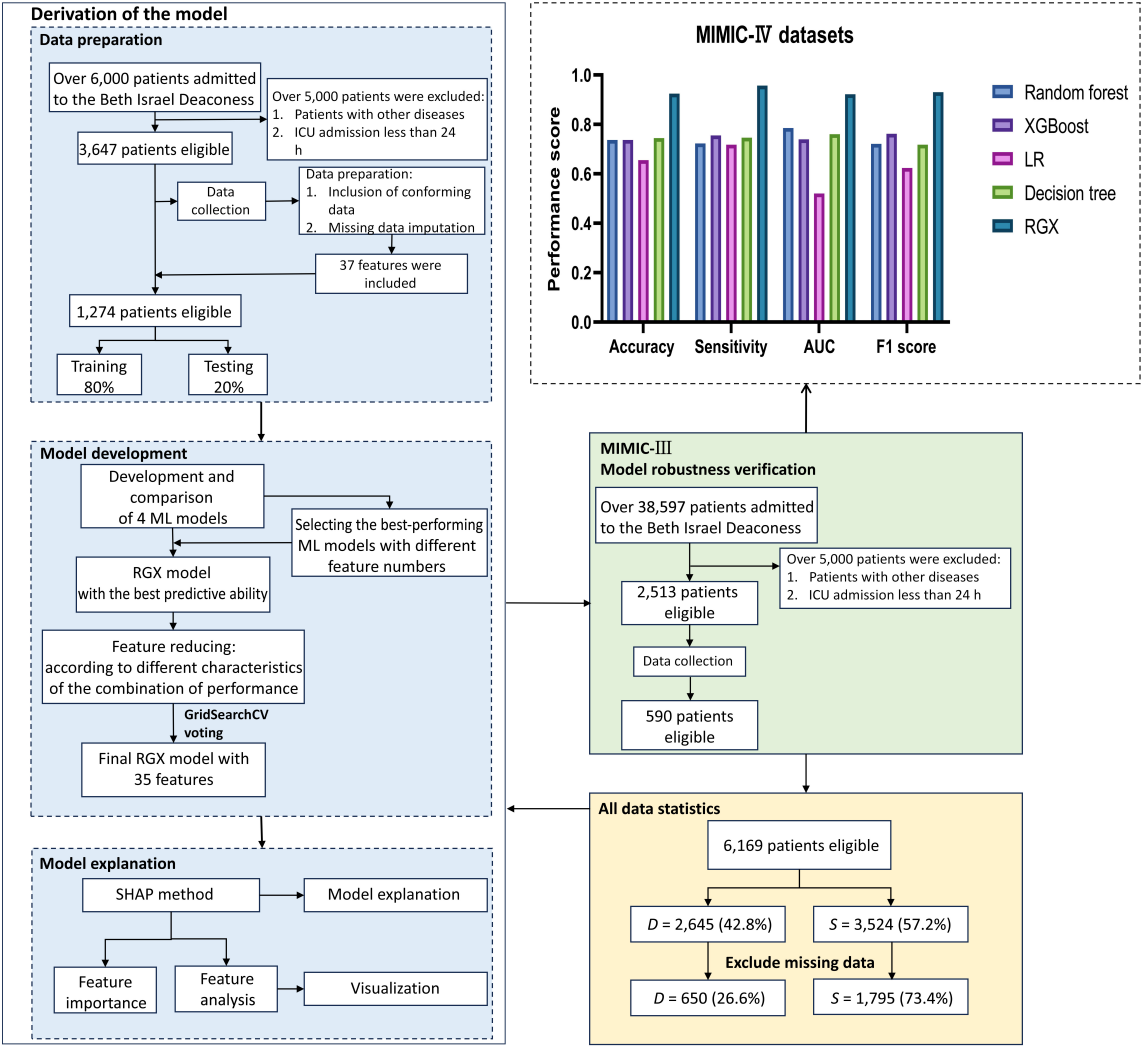
The data filtering process, the model application process, and the Shapley additive explanations (SHAP) plot interpretation section. Statistical analysis is also performed on the Medical Information Mart for Intensive Care IV (MIMIC-IV) and Medical Information Mart for Intensive Care II (MIMIC-III) datasets.

The data were imported into the RGX ensemble model, which was proposed in this study. For different data combinations, the model parameters were adjusted with greater precision to generate Shapley additive explanations (SHAP) plots of various features in the data. The objective was to facilitate the interpretation of the model, assist clinicians in understanding the significance of each feature on the final outcome, and thereby enhance the clinical interpretability of the ensemble model. The specific implementation process is illustrated in Fig. 1.

In the MIMIC-IV database, the composite model showed performance superior to that of the other models and the overall results were satisfactory. Figure 1 illustrates the performance of the RGX model on a range of evaluation metrics when applied to the MIMIC-IV dataset and compared to that of the base machine learning model. The evaluation metrics are divided into 4 categories: accuracy, which indicates the ratio of the number of correctly predicted samples to the total number of samples; sensitivity, which reflects the model’s ability to correctly identify the positive class among all positive class samples; AUC, which measures the model’s classification ability by calculating the area under the receiver operating characteristic curve; and the F1 score, which is used to balance the accuracy with the recall in class-imbalanced data. Different model types are indicated by different colors, with blue indicating the performance of the RGX model across the 4 evaluation metrics, where the results are substantially better than those of the other 4 models.

In particular, the integrated RGX model shows significant improvement in all metrics compared to the most effective base model: a 17.68% improvement in accuracy compared to the most effective decision tree model, a 13.72% improvement in sensitivity compared to the most effective XGBoost model, a 15.85% improvement in AUC compared to the most effective random forest model, and a 16.8% improvement in F1 score compared to the most effective base model, XGBoost. In summary, the RGX model demonstrates excellent performance on all 4 evaluation metrics and a significant improvement in overall effectiveness compared to current base machine learning models. Furthermore, the accuracy has improved by 11.38% compared to that of the current state-of-the-art models [19].

**Fig. 2.**
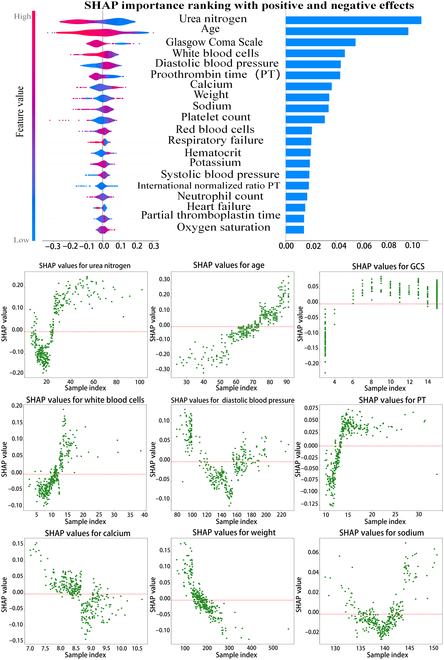
Clinical interpretation of SHAP technology applied to the MIMIC-IV dataset, which indicates that the top 3 factors affecting stroke mortality outcomes are urea nitrogen, age, and Glasgow Coma Scale (GCS). The scatter plots provide further detail on the interpretation of the SHAP value, indicating that when the value is greater than zero, it signifies a positive effect on the prediction result. This feature enhances the predictive value, thereby approximating the positive class label (or a higher numerical prediction).

The integrated model’s decision-making process is complex and opaque. While various evaluation indicators, such as input patient data and output accuracy, can be observed during the experiment, it is challenging to comprehend the model’s judgment and the ranking of the impact of each indicator in the patient data on the patient’s death outcome. Consequently, SHAP technology is employed to elucidate the decision-making process of the integrated model, facilitate the clinical interpretation of the results yielded by the model, and determine the ranking of factors that influence the death outcome of stroke patients, as illustrated in Fig. 2. The 5 factors with the greatest impact on the mortality of stroke patients in MIMIC-IV are urea nitrogen, patient age, Glasgow Coma Scale (GCS), white blood cell count, and blood pressure. The SHAP values of the top 9 factors depicted in Fig. 2 are presented in the figure. The red line represents the 0 baseline, which is used to differentiate the impact of SHAP values on the outcome. A SHAP value greater than zero indicates a positive influence on the outcome.

Higher urea nitrogen level, older age, higher GCS score, higher white blood cell count, and longer prothrombin time are positively correlated with the increased risk of mortality in patients with ischemic stroke, while serum calcium level and body weight are negatively correlated with the risk of mortality. For diastolic blood pressure levels and serum sodium levels, lower or higher levels are associated with an increased risk of mortality.

### Analysis of model generalizability

Given the inherent limitations of clinical data, the quantity and quality of the data may be compromised. For example, the Medical Information Mart for Intensive Care III (MIMIC-III)-extracted stroke patient dataset, which also adheres to the MIMIC-IV data extraction and patient exclusion criteria, was ultimately compiled as the MIMIC-III dataset. The dataset is characterized by a smaller amount of data and a higher prevalence of missing values, which are also highly probable in clinical practice. Accordingly, the MIMIC-III dataset was employed as a supplementary resource to ascertain the model’s generalizability. The MIMIC-III database comprises deidentified health-related data on over 40,000 patients admitted to the intensive care unit (ICU) of the Beth Israel Deaconess Medical Center between 2001 and 2012. Following the application of the aforementioned exclusion criteria, the dataset comprised 2,513 patients. Patients with missing data in excess of 13% and patients with missing variables in excess of 50% were excluded. Due to the considerable number of absent features, a total of 590 patients were selected for model training following the screening process.

In this study, a more straightforward decision tree regression model is employed to predict and supplement the missing values. For each feature with a missing value, the model is trained using the remaining features to predict the missing value for that feature. The discrepancy between the proportions of patients with outcomes of 0 and 1 was addressed through the application of oversampling techniques. The optimal parameters of the model were identified through a grid search, and a variety of feature value combinations were input into the ensemble model. The final output of the ensemble model was determined through a soft voting method within a voting scheme. The model parameters were adjusted with greater precision for different data combinations to generate a SHAP plot of the various features in the data.

**Fig. 3.**
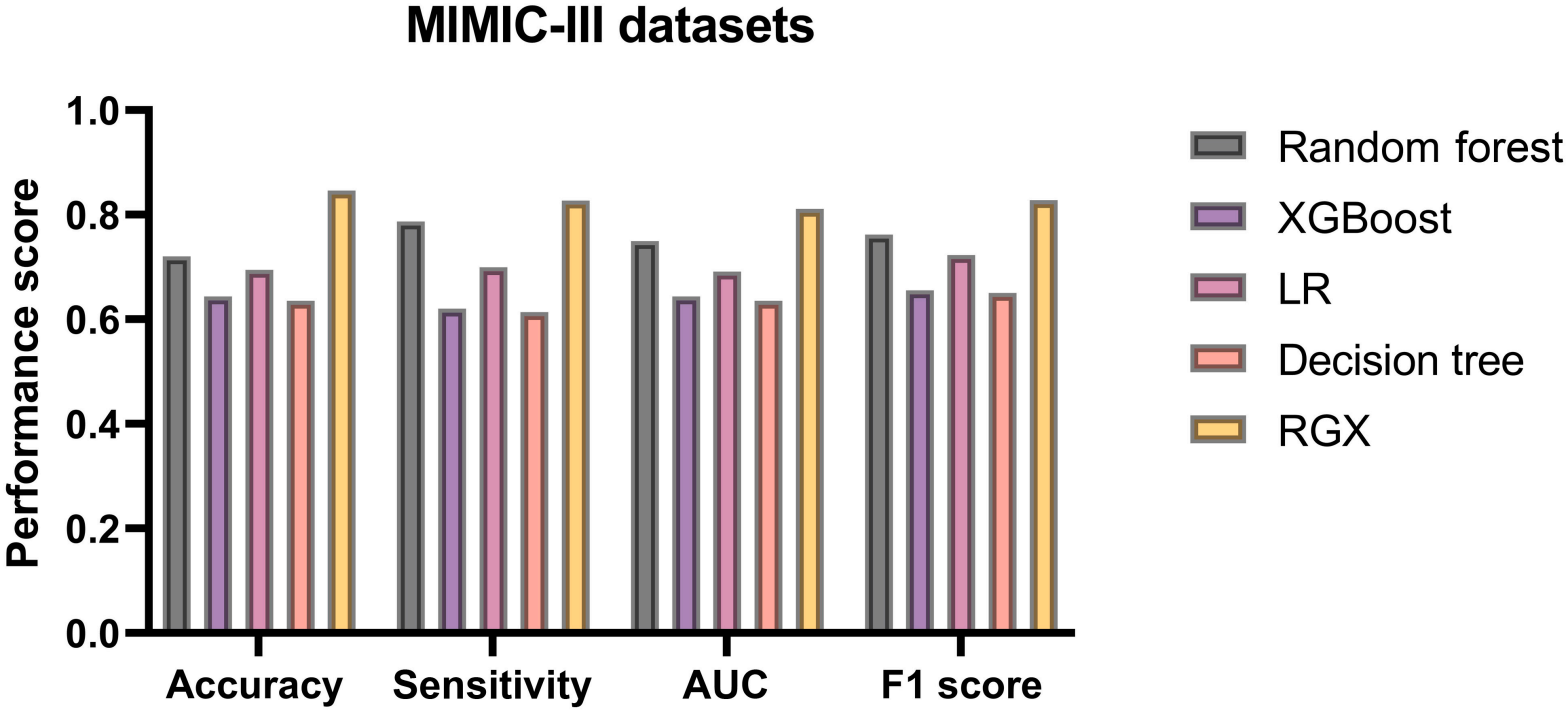
MIMIC-III dataset model performance comparison chart.

Figure 3 shows the performance of the RGX model applied to the MIMIC-III dataset on various evaluation metrics and compares it with that of the base machine learning model. The evaluation metrics are divided into 4 categories: accuracy, sensitivity, AUC, and the F1 score. Each group represents a different model type through different colors, with yellow representing the performance of the RGX model in these 4 evaluation metrics, with substantially better results than the base model.

Specifically, the RGX model performs best in accuracy, with a 12.58% improvement over the best-performing random forest model. In sensitivity, RGX also performs 3.97% better than the better-performing random forest model, indicating that it is better at detecting positive class samples. The RGX model proposed in this study leads the other models in the AUC metric with an improvement of 6.16%, and the model also received the best F1 score, indicating that it performs better in balancing precision and recall.

## Discussion

In this study, 4 machine learning models and an integrated model were evaluated for their ability to predict outcome metrics in stroke patients [20,21]. While the random forest model demonstrated performance superior to that of the basic machine learning model, it was outperformed by the integrated model in this study. The integration of the aforementioned model with the SHAP technique facilitates a comprehensive understanding of the model’s underlying principles, particularly in a clinical context. This integration enables the gradual elucidation of a machine learning model that may otherwise be perceived as opaque.

Notwithstanding the considerable advancements in science, technology, and medicine, clinicians remain unable to accurately ascertain the ranking of physical indicators that affect the mortality of stroke patients and to predict whether the patient will ultimately succumb. Accordingly, this study proposes a comprehensive model and oversamples the dataset due to the imbalance of patient outcomes in public data. Subsequently, 3 machine learning models are integrated, and the optimal parameters of the integrated model are selected through automatic parameter adjustment. The resulting indicators are then optimized in comparison to those of commonly used machine learning models. The integrated model demonstrated an accuracy of 92.18%, an 11.38% improvement over that of the current model utilized for MIMIC-IV stroke patient mortality prediction. The integrated model was subsequently applied to the field of stroke patient prognosis prediction. The SHAP algorithm was employed to ascertain the significance of each predictor in the RGX integrated model with respect to the prediction outcomes, thus furnishing a foundation for clinicians’ diagnosis. Moreover, the SHAP algorithm can ascertain the ranking of factors that influence prognosis, with those at the top of the list exerting a more pronounced impact on patient prognosis. This can be employed to ascertain the factors that exert the greatest influence on disease prognosis in a clinical context. The SHAP algorithm can be employed to ascertain the factors most closely associated with patient death outcomes.

In comparison to the existing machine learning models utilized for disease prediction, the machine learning model proposed in this study has demonstrated a notable enhancement in prediction performance. Moreover, when integrated with the prevailing SHAP technology, the model is capable of not only forecasting a prognosis but also elucidating the prediction outcomes from a clinical standpoint in a comprehensive manner. Moreover, the model is capable of accurately identifying the relative importance of influencing factors on outcome indicators, which is consistent with clinical data. This demonstrates the efficacy of the comprehensive model in predicting disease outcomes. Furthermore, the model enables rapid and precise evaluation of stroke severity and potential prognosis by clinicians, a capability that is not yet fully operational in clinical practice. The integrated model offers a novel screening method. The application of the RGX integrated model proposed in this study to MIMIC-IV events resulted in an increase in accuracy by 17.68% compared to that of the basic model used for prediction. Furthermore, in comparison to the most effective random forest model within the basic model (78.50%), the AUC increased to 92.20%, the sensitivity increased by 13.72%, and the F1 score increased by 16.8%. In conclusion, the integrated model has markedly enhanced its capacity to predict mortality outcomes in stroke patients.

The model can be applied to different clinical areas. Regardless of whether the public dataset MIMIC-IV extracted a large number of datasets or MIMIC-III extracted a small number of datasets, a comparison can be made with the traditional machine learning indicators, which have undergone a significant improvement. Furthermore, the integrated model can be used to complete the prediction of the ending labels with fewer indicators, which indicates that the model is highly versatile and robust.

It should be noted, however, that the study is not without limitations. First, the study was a retrospective analysis of patient data collected from 2008 to 2019. This limits the ability to elucidate the effects of all clinical variables on stroke patient outcomes. Further studies are needed to validate the effect of targeting all clinical variables on the model’s ability to predict patient outcomes. Second, it was not feasible to ascertain whether unmeasured variables, in addition to those already considered, exerted an influence on stroke prognosis and to what extent. Factors with a high degree of clinical acceptance were selected for training and testing of the model, and other factors have not yet been included clinically. Therefore, it is not possible to specifically assess the impact of factors other than those selected. Moreover, the data utilized in this experiment were derived from disparate versions of a single public dataset. Consequently, the outcomes of the final integrated model may not be directly applicable to the prediction of other diseases. To achieve optimal accuracy, the model’s parameters must be adjusted according to the characteristics of different diseases and other pertinent factors. Nevertheless, the model’s efficacy in forecasting the prognosis of stroke patients indicates that it may be adaptable to the prediction of diagnoses for other diseases, contingent on the requisite adjustments to specific parameters.

The model has a wide range of potential future applications. It can be combined with electronic medical records (EMRs) to predict the type of disease based on the patient’s scores on various indicators, as well as to identify the most relevant influencing factors on outcome. This provides a basis for doctors’ diagnosis and treatment. Concurrently, the model can be utilized for the prediction of other diseases, based on the impact of the disease’s characteristics to ascertain the likelihood of the disease manifesting. As EMRs become more prevalent, it is possible to combine the 2 systems to extract the necessary feature values and other influencing factors through EMR extraction technology. These can then be integrated with the model to provide diagnostic and treatment advice, aiding physicians in their diagnosis and treatment decisions. The model has a vast range of potential applications, with prospects for further development that are limitless.

## Materials and Methods

### Materials

#### Data Processing

The datasets MIMIC-III and MIMIC-IV used in this study were obtained from the PhysioNet website (https://physionet.org/) with permission to download. The MIMIC-III database collects data on patients admitted to the ICU at the Beth Israel Deaconess Medical Center in the United States from 2001 to 2012. The database contains deidentified health data on more than 40,000 ICU patients, including patient demographics (e.g., age and gender), vital signs (e.g., heart rate and blood pressure), laboratory results (e.g., blood and urinalysis), medical diagnoses (ICD-9 compliant), medication records, medical procedures and treatment plans, ICU care records and discharge records, imaging and test results, and hospital charges and billing data. The MIMIC-IV database represents an enhanced iteration of the MIMIC-III database, incorporating data collected from 2008 to 2019 from the ICU at the Beth Israel Deaconess Medical Center. It encompasses a more expansive and detailed representation of ICU patients, encompassing over 70,000 individuals.

#### Dataset Construction

This paper takes the MIMIC-IV database as an example for data collection, and the MIMIC-III database is operated in the same way. Firstly, to determine the study population, the aim of this study is to investigate the prognosis of stroke patients, making it necessary to screen stroke patients admitted to hospitals. The database was searched for the diagnostic codes corresponding to stroke, which were divided into ICD-9 and ICD-10 (only ICD-9 in MIMIC-III), and all patients were screened based on these codes. There are 2 types of patient identifiers: subject_id and hadm_id. subject_id is a unique identifier of each patient in the MIMIC database. There is only one subject_id per patient, even if the patient has multiple hospitalizations or ICU records. hadm_id is unique for each admission, and a patient may have more than one hadm_id. For patients with multiple admissions, the first admission records are sorted by the time of admission, and the first admission records are selected to create the dataset, which filters out the patients for use in creating the dataset, ensuring that the patient information is unique and avoiding duplication of information.

Once the patient information utilized to generate the dataset had been identified, the patient’s subject_id was employed to filter the requisite data. Subsequently, the pgAdmin4 software was utilized to filter the data through the use of Structured Query Language, with the objective of identifying the data employed in the construction of the dataset. Ultimately, 35 eigenvalues and one ending variable were filtered, and the eigenvalues included the following: The variables included in the dataset were as follows: gender, age, body mass index, body weight, systolic blood pressure, diastolic blood pressure, oxygen saturation, GCS, smoking status, hyperlipidemia, diabetes, atrial fibrillation, coronary heart disease, heart failure, and other outcomes. The following conditions were identified: diabetes, atrial fibrillation, coronary heart disease, heart failure, pneumonia, respiratory failure, renal disease, sepsis, white blood cells, red blood cells, platelets, neutrophils, hematocrit, activated partial thromboplastin time, and prothrombin time. The variables used for dataset construction included international normalized ratio, D-dimer, blood urea nitrogen, bicarbonate, sodium, calcium, potassium, triglyceride, mechanical thrombectomy, alteplase, and the ending indicator dod_flag. The final number of patients included in MIMIC-IV was 3,647, while the number of patients included in MIMIC-III was 2,513. This indicates that the construction of the database input into the model was completed, thereby ensuring the uniqueness of patient information and the accuracy of the dataset.

### Cohort Description

The training data for this study were derived from MIMIC-IV, a comprehensive database that is accessible to researchers at no cost. The database contains health-related data on more than 40,000 inpatients in the ICU of the Beth Israel Deaconess Medical Center. The database comprises 26 tables that record basic patient information. The present study employed the database to investigate the characteristics and outcomes of stroke patients. The database was queried for patients with a diagnosis of ischemic stroke according to the International Classification of Diseases (ICD-9 and ICD-10). The exclusion criteria were as follows: (a) For patients who were admitted and discharged from the ICU on multiple occasions, only the initial admission was included in the analysis. (b) Patients with missing data representing more than 13% of the total were excluded from the study. Following the application of the aforementioned clinical indicators and exclusion criteria, a number of indicators were selected from the MIMIC-IV database: (a) demographic and vital signs indicators: age, gender, weight, admission systolic blood pressure and admission diastolic blood pressure, and smoking history; (b) clinical scores at admission: the GCS was also considered; and (c) complications (undesired side effects during medical treatment): The following comorbidities were considered: hypertension, diabetes, coronary heart disease, hyperlipidemia, atrial fibrillation, heart failure, respiratory failure, pneumonia, paraplegia, kidney disease, and sepsis. (d) Additionally, laboratory tests were analyzed, including white blood cells, red blood cells, platelets, hemoglobin, sodium, serum creatinine, triglycerides, low-density lipoprotein cholesterol, blood sugar, and fibrinogen. (e) A record was kept of all previous medication history, including antiplatelet drugs, anticoagulants, and lipid-lowering drugs.

### Experimental and technical design

The training set is fed into 5 classification models that are commonly used in machine learning. Based on the results obtained, 3 of the relatively better models are selected for model integration. The 3 models with the best performance of the base model are taken, namely, the random forest model, the gradient boosting model [22], and the decision tree model [23]. The RGX is integrated using these 3 models.

The random forest [24] model is essentially an extension of the decision tree model. A decision tree model is a treelike structure that classifies data using decision rules at its nodes to create different leaf nodes, ultimately achieving classification. Its decision formula is

$$I\left(s,t\right)=\frac{\left|D_{s}\right|}{\left|D\right|}I\left(D_{s}\right)+\frac{\left|D_{t}\right|}{\left|D\right|}I\left(D_{t}\right)-I\left(D\right)$$ (1)

$$H\left(x\right)=-\sum P_{i}*\log\left(P_{i}\right),\;i=1,2,\ldots ,n\;$$ (2)

*I*(*s*,*t*) is the information gain of a split, where |*D*| is the size of the parent node before the split, and |*D*_1_| and |*D*_2_| are the sizes of the left and right child nodes after the split, respectively. Additionally, *P*(*D*) and *P*(*D*_1_), *P*(*D*_2_) represent the impurity measures (e.g., Gini impurity or entropy) of the parent node and the child nodes, respectively.

This measure is also known as entropy. Constructing the decision tree using the highest entropy value yields the best results. Gradient boosting is a machine learning method that builds a strong predictor by combining multiple weak predictors. The core idea is that each subsequent predictor corrects the errors of the previous predictor, gradually improving the model’s accuracy and generalization ability. First, a constant predictor $f_{0}\left(x\right)=\gamma$ is trained, where *γ* is the mean target value of the training data. The prediction from the previous step is $f_{t-1}\left(x\right)$, and the residual for each training sample is $r_{t}=y-f_{t-1}\left(x\right)$, where *y* is the true target value and *t* is the iteration number. A weak predictor $h_{t}\left(x\right)$ is then fitted to predict the residuals. The step size controls the contribution of the predictor. Then, the predictor is updated as $f_{t}\left(x\right)=f_{t-1}\left(x\right)+a_{t}h_{t}\left(x\right)$. The final predictor $f_{T}\left(x\right)$ is the weighted sum of all of the predictors. The XGBoost model’s hyperparameter is optimized by scale_pos_weight = (np.sum(*y* == 0)/np.sum(*y* == 1)). In this study, due to the imbalance between positive and negative labels, this parameter is used to balance the weights of positive and negative labels.

To evaluate the performance of the models, this study used accuracy, recall, precision, and F1 score. The definitions are as follows:

$$\text{Precision}=\frac{\mathrm{TP}}{\mathrm{TP}\;+\;\mathrm{FP}},\;F1\;\text{score}=\frac{2\;\times \;\text{Precision}\;\times \;\text{Recall}}{\text{Precision}\;+\;\text{Recall}}$$ (3)

$$\text{Sensitivity}=\frac{\mathrm{TP}}{\mathrm{TP}\;+\;\mathrm{FN}},\text{specificity}=\frac{\mathrm{TN}}{\mathrm{FP}\;+\;\mathrm{TN}}$$ (4)

where TP, TN, FP, and FN represent true positives, true negatives, false positives, and false negatives, respectively. Figure 3 shows the prediction performance for the 2 different labels by the 5 models used in this study: the proposed RGX model and the random forest, XGBoost, decision tree, and LR models.

Due to the significant disparity between the number of labels 0 and 1, oversampling was used to balance the outcome indicators. By randomly replicating samples of the minority class to increase their quantity, the dataset was balanced, increasing the model’s ability to recognize the minority class. The final output of the RGX model was generated using hard voting. Since the 3 base models might produce different results for the same input data, a voting method was employed to determine the final output of the RGX model. There are 2 types of voting: soft voting and hard voting. Soft voting considers a probability value instead of the final result of each algorithm. It takes a weighted average of these probabilities. For example, if model A assigns a probability of *a* to class 1, model B assigns a probability of *b* to class 1, model C assigns a probability of *c* to class 2, and model D assigns a probability of *d* to class 1, then the average probability for class 1 is

$$p=\frac{1}{3}\left(a+b+d\right)$$ (5)

The probability for class 2 is *c*. If the average probability for class 1 is greater than the probability for class 2, then the final prediction would be class 1. In hard voting, the experimental data are input into 4 machine learning models, and the final result is determined by majority rule. If 3 out of 4 models predict label 1 and 1 model predicts label 0, the final classification, after hard voting, is label 1. This method follows the principle of majority rule. Since this study predicts clear class labels, soft voting is used for model ensemble.

**Fig. 4.**
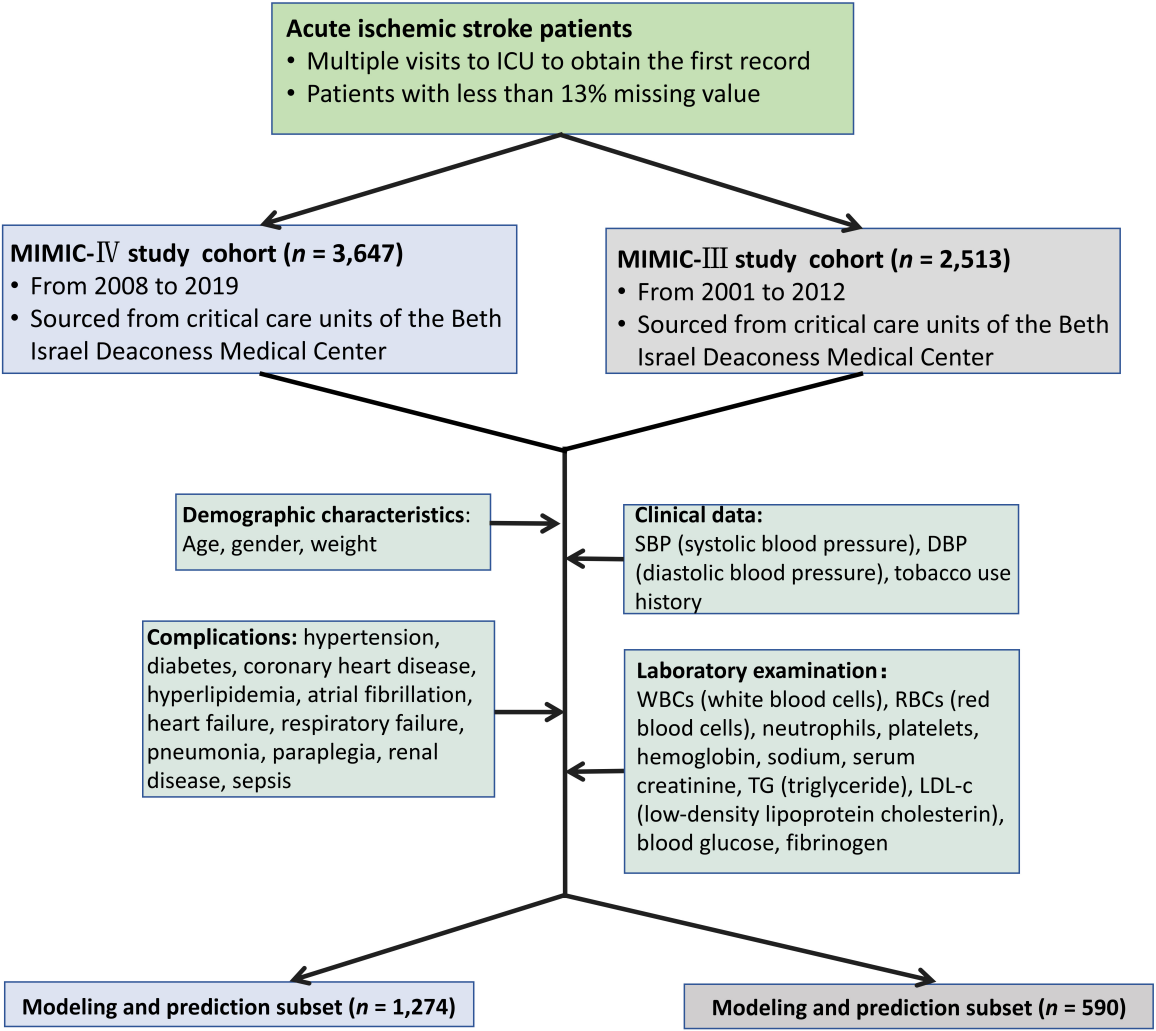
MIMIC-IV and MIMIC-III patients’ selection process.

In the RGX model, the parameters are selected via the GridSearch method. A variety of combinations of hyperparameters are provided, and the appropriate hyperparameters are selected using scikit-learn’s GridSearchCV method. The integration process of the model is illustrated in Fig. 4.

The training data and validation data were input into the corresponding parameters of the random forest model, XGBoost model, LR model, and decision tree model, respectively, in order to identify the common points of their models. Firstly, with regard to the sensitivity of the 4 models, it was found that the random forest, XGBoost, and decision tree models exhibited higher sensitivity than the other base models. Secondly, with regard to accuracy, the 3 base models that were used to integrate the RGX model also demonstrated superior performance. Ultimately, an analysis of the AUC for the selected features revealed that the 3 base models also demonstrated the most optimal performance. Consequently, the 3 models were employed to integrate the RGX model. The parameters of the model, contingent on the type of input data, the dynamic adjustment of the model parameters, and the weight of the base model in the integrated model, can enhance the accuracy of the output and optimize the performance of the evaluation indexes.

LR [25] is a generalized linear regression model that assumes that the data follow a Bernoulli distribution. It maximizes the likelihood function to determine parameters using gradient descent, achieving binary classification of the data [26].

$$y=\frac{1}{1+{\mathrm{e}}^{-f\left(x\right)}}=\frac{1}{1+e^{-w^{T}x}}$$ (6)

In the LR model, *x* represents the feature inputs, *w* denotes the parameters, and *y* is the output. The model’s key advantages include its quick training capability, even on large datasets; its strong performance for binary classification problems; and its ability to understand the relationship between features and the outcome [27]. The parameter for the LR model is set to max_iter = 10,000.

XGBoost, short for extreme gradient boosting, is an optimized distributed gradient boosting library designed to be highly efficient, flexible, and portable. It integrates multiple tree models into a strong classifier [28], making it suitable for binary classification problems in this study. In the XGBoost model, features are sequentially input into each decision tree. Each decision tree’s corresponding nodes have associated prediction weights. These weights are summed across all decision trees to obtain the final prediction result, with the maximum value being the final prediction output. The parameter optimization for the XGBoost model is set to scale_pos_weight = (np.sum(*y* == 0) / np.sum(*y* == 1)). By setting scale_pos_weight to this calculated ratio, the XGBoost model can effectively balance the labels, leading to more accurate predictions for the minority class in this study.

A decision tree is a treelike structure used for classification and regression tasks. It produces results as leaf nodes following a series of tests [23]. The structure of a decision tree comprises 3 main components: the root node, nonleaf nodes with branches, and leaf nodes [12].

The random forest model extends the decision tree algorithm, by combining multiple decision trees to create a “forest”, with each tree independently trained [29]. It is an ensemble learning model based on the bagging (bootstrap aggregating) strategy, incorporating sample randomness and feature randomness.

By combining the results of multiple decision trees, the random forest model achieves more accurate and reliable predictions than individual trees alone [30]. The process of random forest involves drawing *n* samples with replacement from the original dataset to create new training subsets, repeating this process *m* times to create different training subsets for trees, training the decision trees on these subsets, and using bagging (voting) to create the final outcome. The random forest model is set with n_estimators = 100 and random_state = 42.

### SHAP technique

**Fig. 5.**
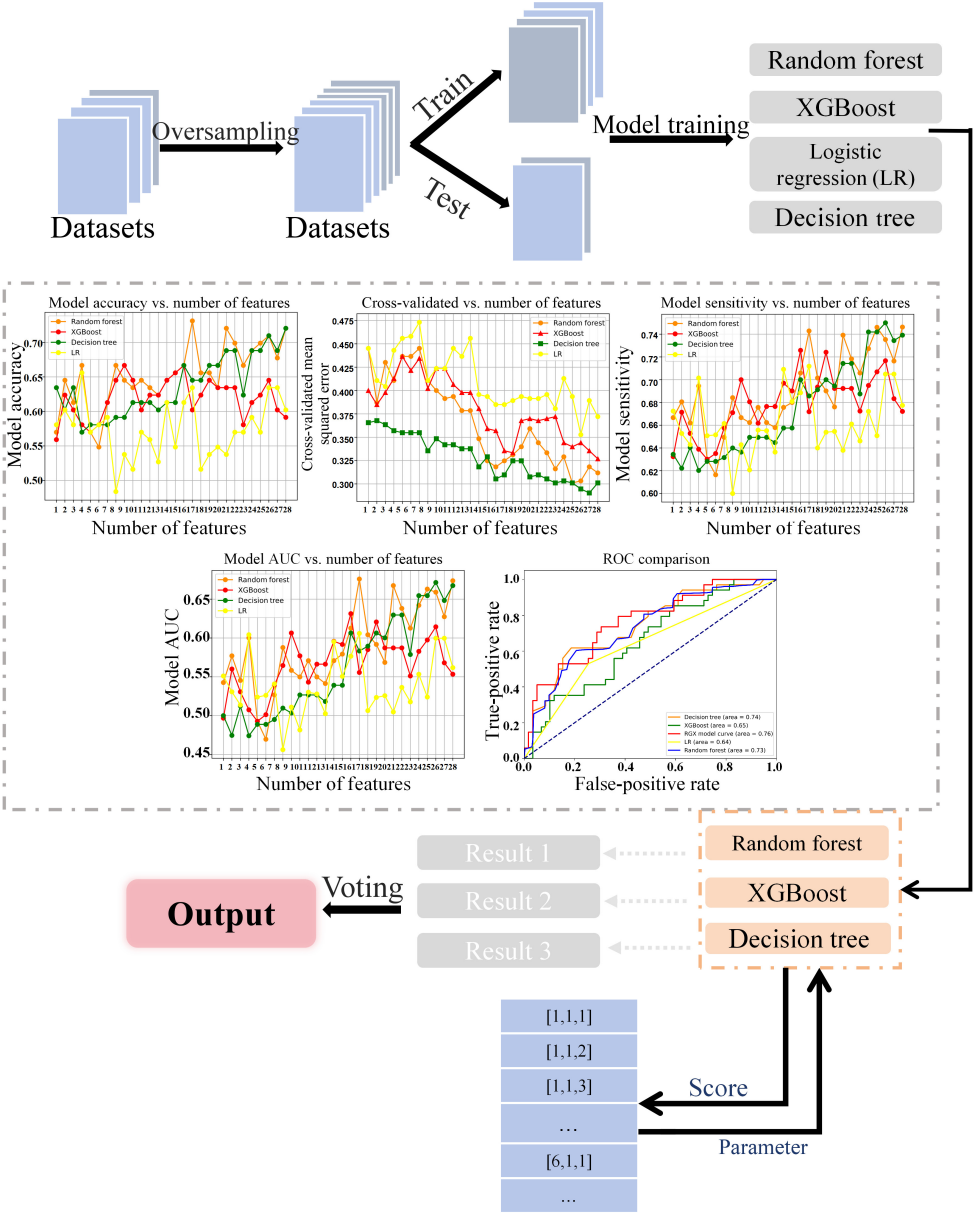
The specific workflow of the RGX integrated model and the impact of varying the number of input features on the performance of 4 machine learning models under 5 indicators.

**Fig. 6. F6:**
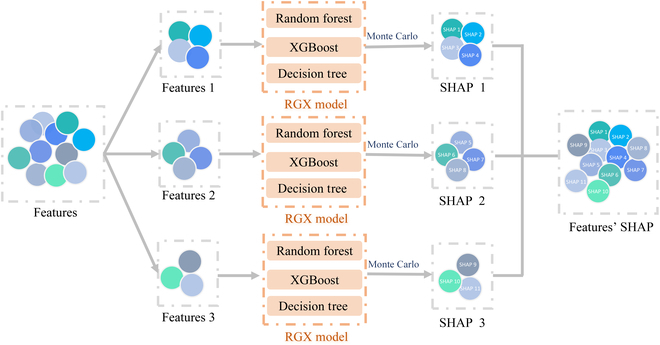
Application of SHAP technology, which determines the importance of features by calculating the marginal contribution of all possible combinations of features. This output generates a ranking of the relative importance of the influencing factors in the model.

SHAP [31,32] plots represent a sophisticated model interpretation tool that can be utilized to elucidate the underlying principles that inform the predictions generated by machine learning models. This method provides not only an overall explanation but also a detailed explanation of individual predictions, thus assisting clinicians in comprehending the principles of model application. The specific principles of SHAP technology are illustrated in Figs. 5 and 6.[Fig F6]

The generation and presentation of SHAP values for various features in a visual format facilitates comprehension of the overall importance of each feature within the model on the actual dataset. This can be accomplished by generating importance maps. The influence of each feature value on the model output can be illustrated by a distribution, with the feature values represented by color (red for high and blue for low). This allows for the clear identification of whether high or low feature values are beneficial or detrimental to the outcome measure [33].

The application of SHAP graph analysis provides a means of elucidating the decision-making process of the integrated model in this study. It offers a rationale for the black-box model of machine learning in the context of clinical application. Furthermore, it enables the degree of contribution of each feature in the model prediction to the prediction results to be quantified. The approach is based on the principles of cooperative game theory, wherein the input features are regarded as participants and the prediction outcomes are viewed as gains. Subsequently, the contribution of each feature to distinct prediction outcomes is quantified. First, a given set of parameters is divided into distinct subsets of features. For each permutation, the marginal contributions of the feature values are calculated, weighted, and averaged. The weights utilized in the calculation are contingent upon the influence of other features, taking into account both the frequency of occurrence of the feature in question and its exclusion from the aforementioned permutation. The result of the weighted average is subsequently employed as a SHAP value, which signifies the mean marginal contribution of the feature to the model prediction and the extent to which each feature contributes to the model prediction can subsequently be interpreted based on this value. The application of the SHAP technique to the RGX integration model in this study has the potential to elucidate the underlying reasons for this result in the clinical setting and to enhance the model’s generalizability.

### Statistical analysis

The prognosis of stroke is contingent upon a series of clinical indicators, including the presence and severity of patient complications. The use of an integrated model to predict the prognosis of stroke patients and to analyze the impact of each indicator on outcome variables using the SHAP technique can assist clinicians in the screening of patients for appropriate follow-up treatment while simultaneously ensuring the rights and interests of patients. The integrated model can be employed in clinical diagnosis at a subsequent stage to assist physicians in determining the severity of a patient’s condition and whether or not to proceed with a series of subsequent treatments through the use of different indicators. Moreover, the model can be applied to patients with disparate outcome indicators. Subsequent predictions can be made using the integrated model in the context of patients with varying diseases. This approach is widely applicable and is anticipated to become a valuable technical tool for assisting physicians in the diagnosis and prediction of diseases in their later stages.

## Data Availability

The data utilized in this study were obtained from the MIMIC public dataset, with record ID 60121385.
