# Supplementary figures and images for: RGX Ensemble Model for Advanced Prediction of Mortality Outcomes in Stroke Patients
Source: BME Front. 2024 Nov 26;5:0077. doi: 10.34133/bmef.0077 (PMC11588983; doi:10.34133/bmef.0077)

## SUPPLEMENTARY MATERIALS

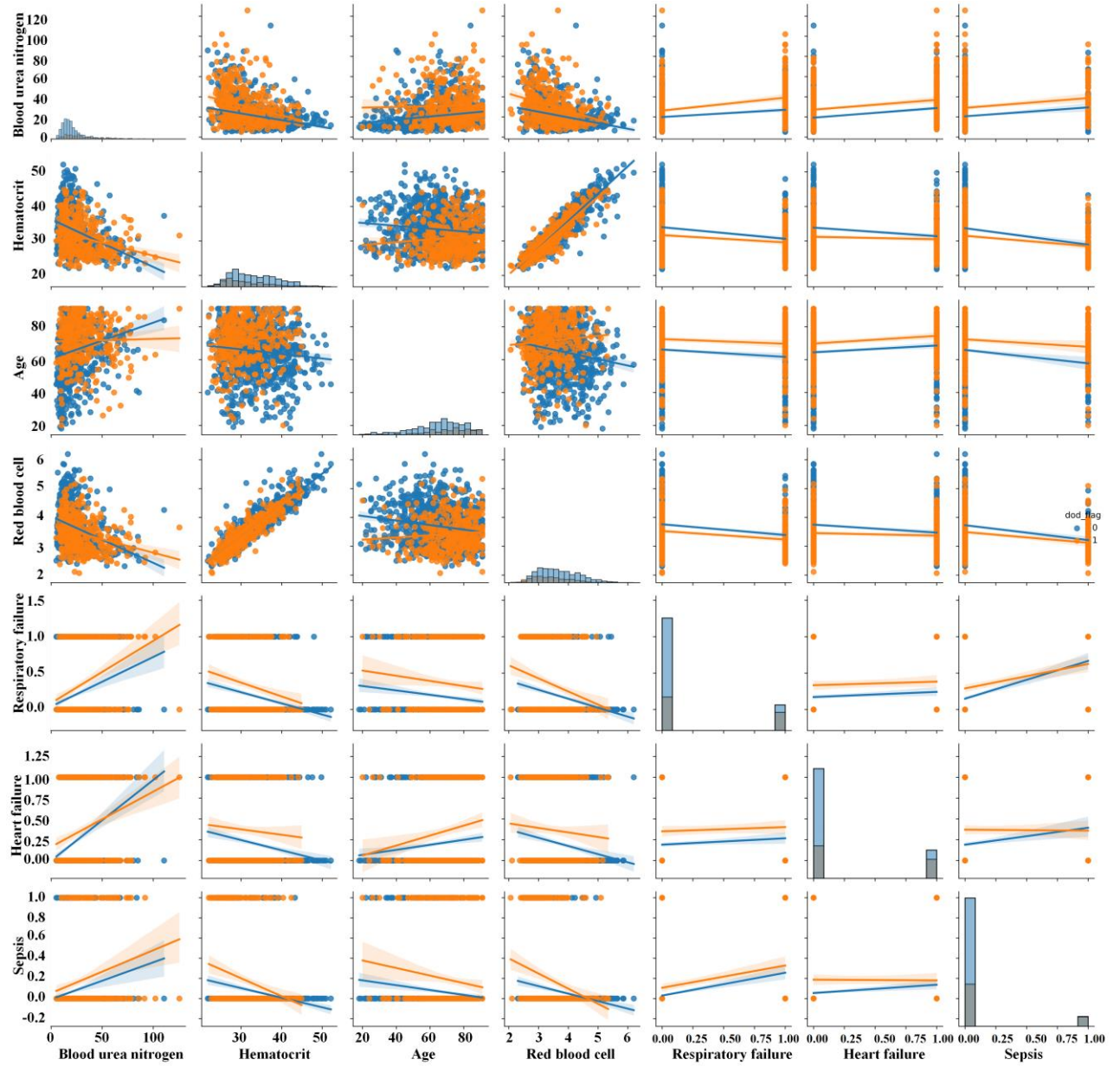

Figure S1: Pair plots analysis of the MIMIC-IV dataset

Supplement: Supplementary 1 — Fig. S1 [file bmef.0077.f1.pdf]
